# TRIM37 Promotes Pancreatic Cancer Progression through Modulation of Cell Growth, Migration, Invasion, and Tumor Immune Microenvironment

**DOI:** 10.3390/ijms23031176

**Published:** 2022-01-21

**Authors:** Tuyen Thi Do, Chun-Chieh Yeh, Guo-Wei Wu, Chia-Chen Hsu, Hung-Chih Chang, Hui-Chen Chen

**Affiliations:** 1 International Master’s Program of Biomedical Sciences, College of Medicine, China Medical University, Taichung 404328, Taiwan; cherryblossomhmu@gmail.com; 2 Department of Laboratory Hematology, Hanoi Medical University, Hanoi 11520, Vietnam; 3 Department of Surgery, School of Medicine, China Medical University, Taichung 404328, Taiwan; b8202034@gmail.com; 4 Organ Transplantation Center, Department of Surgery, China Medical University Hospital, Taichung 404327, Taiwan; 5 Department of Surgery, Asia University Hospital, Taichung 413505, Taiwan; 6 Graduate Institute of Biomedical Sciences, China Medical University, Taichung 404328, Taiwan; j22038572@gmail.com (G.-W.W.); b90330111@hotmail.com (H.-C.C.); 7 Department of Biological Science and Technology, China Medical University, Taichung 404328, Taiwan; ghsu612@gmail.com; 8 Department of Microbiology and Immunology, School of Medicine, China Medical University, Taichung 404328, Taiwan; 9 Research and Development Center for Immunology, China Medical University, Taichung 404328, Taiwan

**Keywords:** TRIM37, pancreatic cancer, immune microenvironment

## Abstract

TRIM37 dysregulation has been observed in several cancer types, implicating its possible role in tumorigenesis. However, the role of TRIM37 in pancreatic cancer progression remains unclear. In the present study, we observed that TRIM37 knockdown resulted in reduced proliferation, clonogenicity, migration, and invasion ability of pancreatic cancer cells. Furthermore, an in vivo study using an orthotopic syngeneic animal model further confirmed that reduced expression of TRIM37 in cancer cells suppressed tumor growth in vivo. Moreover, in mice bearing TRIM37 knockdown pancreatic cancer cells, the proportion of CD11b^+^F4/80^+^MHCII^low^ immunosuppressive macrophages was significantly reduced in tumor milieu, which might be due to the regulatory role of TRIM37 in cytokine production by pancreatic cancer cells. Collectively, these findings suggest a key role of TRIM37 in promoting pancreatic cancer progression.

## 1. Introduction

Pancreatic cancer is the fourth leading cause of mortality in Western countries and has been predicted to become the second leading cause of cancer-related deaths in a decade [1]. Pancreatic ductal adenocarcinoma (PDAC) stems from the exocrine pancreas and accounts for 95% of pancreatic cancers [2]. *KRAS* is one of the most frequently mutated oncogenes in PDAC, colorectal cancer, and non-small cell lung cancer [3]. Despite tremendous improvements in diagnostics and treatment, cancer therapy remains a challenging task for health professionals because of the advanced and metastatic stages and resistance to diverse treatments. The five-year survival rate of patients with pancreatic cancer is lower than 4% [4]. The immune system plays a critical role in the pathogenesis of pancreatic cancer. Previous studies have shown that only KRAS mutations are not sufficient for the development of PDAC in rodents [5,6]. Inflammatory insults that result in tissue damage and genetic instability are associated with disease progression [7,8]. In addition, pancreatic cancer cells can produce various cytokines and chemokines to promote inflammation and establish an immunosuppressive microenvironment [9,10].

Tripartite motif-containing 37 (TRIM37) is a member of the TRIM family of proteins comprising the RING finger, B-box, and coiled-coil domain at the C-terminal and N-terminal containing DES (aspartate-glutamate-serine), nuclear localization signals (NLS), and Meprin and TRAF-homology domain (MATH) [11,12,13]. Patients with Mulibrey nanism, which is the consequence of autosomal recessive mutations in the gene encoding the TRIM37 protein, have increased the risk of both benign and malignant tumors, vascular lesions, and impaired organ development [14]. TRIM37 has been implicated in playing a role in the tumorigenesis of breast cancer, human glioma, non-small lung cancer, and gastric cancer [15,16,17,18]. The activation of Ras suppresses the expression of the Fas gene, thereby reducing Fas ligand-induced apoptosis [19]. Gazin et al. has demonstrated that TRIM37 is required for Ras-mediated epigenetic gene silencing [20]. A recent study revealed that TRIM37 is an oncoprotein that facilitates the silencing of tumor suppressors by functioning as a histone H2A ubiquitin ligase. The overexpression of TRIM37 increases the ubiquitination of H2A, leading to increased activation of transforming growth factor (TGF-β)-signaling pathway to promote renal cell carcinoma [21].

Among the TRIM family proteins, TRIM37 is the only member containing the MATH domain; thus, it can interact with proteins containing the TNF-receptor-associated factor (TRAF) domain. Indeed, a study has shown that TRIM37 could interact with members of the TRAF protein family and the TRAF-binding protein I-TRAF/TANK in vitro [22]. TRAFs are critical components in the regulation and recruitment of effector proteins and other signaling complexes; therefore, they can activate the subsequent components, regulate the subcellular localization of the complexes, and control the degree of response [23]. TRAF-associated signaling pathways are associated with the activity of NF-κB, MAPKs, and interferon-regulatory factors [24]. TRIM37 has been demonstrated to participate in the polyubiquitination of TRAF2 at the position of lysine 63, eventually leading to the activation of the NF-κB pathway in non-small cell lung cancer [25]. Under genotoxic stress, TRAF6 could bind to TRIM37 and promote the monoubiquitylation of NEMO, which could lead to increased cisplatin resistance in esophageal cancer through the activation of NF-κB signaling [26]. The activation of KRAS in PDAC enhances the production of various cytokines and chemokines, which alter the immune microenvironment to facilitate cancer progression. Interestingly, a recent study showed that TRIM37 enhances the ubiquitination of TRAF6, and the knockout of TRIM37 decreases the proinflammatory cytokine production by macrophages during viral infection [27]. In this study, we aimed to investigate whether TRIM37 could facilitate pancreatic cancer progression by modulating cell proliferation, clonogenicity, migration, invasion, and the tumor immune microenvironment through the regulation of cytokine production in pancreatic cancer cells.

## 2. Results

### 2.1. Cell Viability Was Decreased in TRIM37 Knockdown Pancreatic Cancer Cell Lines In Vitro

To investigate the functional role of TRIM37 in pancreatic cancer, the mouse pancreatic cancer cell line, Pan18-GFP-Luc (Pan18), and human pancreatic cancer cell lines, BxPC-3 and PANC-1, were infected with lentivirus bearing shTRIM37 plasmid to knockdown the expression of TRIM37. The protein expression of TRIM37 in pancreatic cancer cell lines was detected by western blotting. The results illustrated that the protein expression of TRIM37 in TRIM37 knockdown pancreatic cell lines was decreased compared with that in the WT and shLuc control groups. The knockdown efficacy was approximately 50% (Supplementary Figure S1).

**Figure 1:**
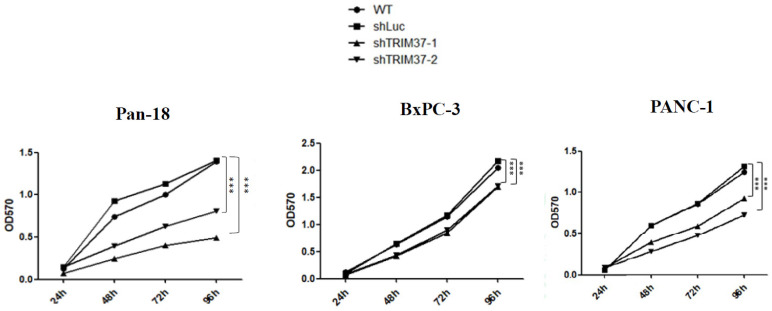
Knockdown of TRIM37 decreased pancreatic cancer cell viability. An MTT assay was performed to measure the viability of cells at different time points. The numbers represent the mean absorbance at 570 nm ± SEM (*n* = 3). Statistical significance was determined using two-way ANOVA with Bonferroni’s post-hoc test (*p* < 0.001: *** compared to shLuc). WT: wild type cells; shLuc: cells bearing shLuc plasmid; shTRIM37-1: cells bearing shTRIM37-1 plasmid; shTRIM37-2: cells bearing shTRIM37-2 plasmid.

TRIM37 can upregulate many signaling pathways, including NF-κB, PI3K/Akt, and Wnt/β-catenin [12]. Since these pathways are involved in cell proliferation and survival, we examined whether TRIM37 facilitates pancreatic cancer cell proliferation. After confirming the protein expression level in various cell lines, the MTT assay was conducted to explore the downstream effect of TRIM37 knockdown on pancreatic cancer viability. As shown in Figure 1, there was no obvious difference in the proliferation between the WT and shLuc control groups. However, when the expression of TRIM37 in cancer cells was knocked down, cell viability was significantly reduced (Figure 1).

### 2.2. Cell Migration and Invasion Were Suppressed in TRIM37 Knockdown Pancreatic Cancer Cell Lines In Vitro

TRIM37 has been reported to promote the migration and invasion of glioma cells in vitro [16]; therefore, we investigated whether TRIM37 could enhance the migration and invasion of pancreatic cancer cells. We examined the effect of TRIM37 knockdown on pancreatic cancer cells’ migration and invasion ability using wound healing assay and Matrigel invasion assay.

**Figure 2:**
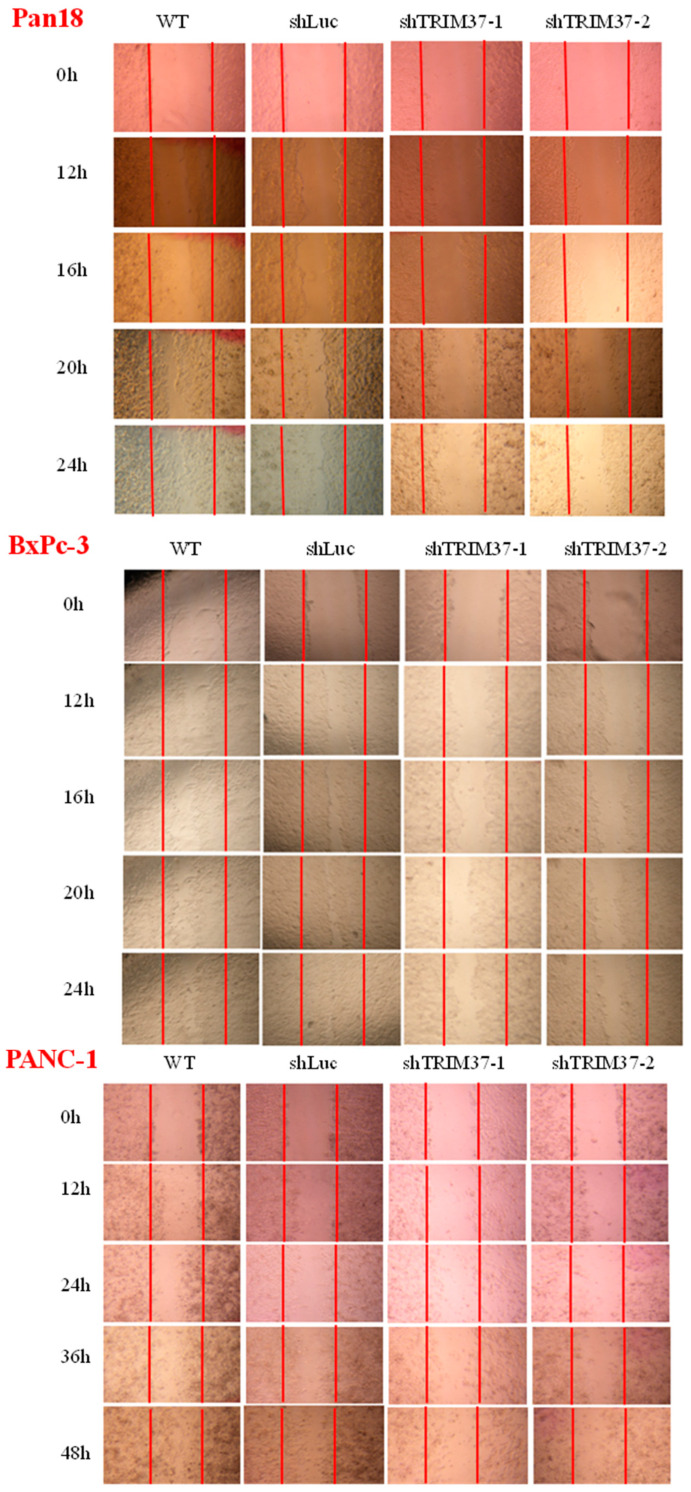

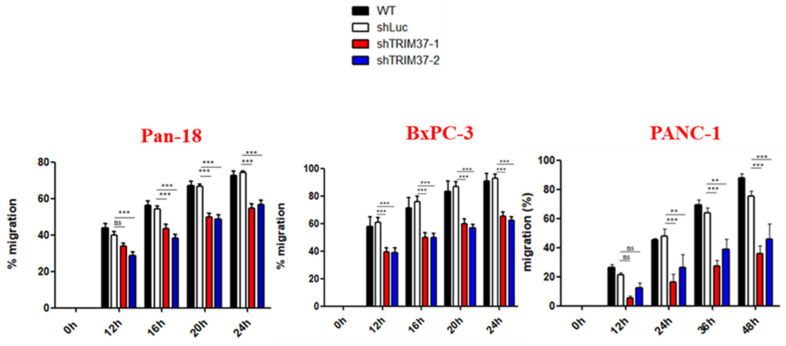
The knockdown of TRIM37 reduced pancreatic cancer cell migration ability. A wound healing assay was conducted to measure pancreatic cancer cell migration ability of Pan18, BxPC-3, and PANC-1 cells. Representative images of different pancreatic cancer cell monolayers (Original magnification, ×10) and quantification of wound closure are presented. Numbers represent the mean wound closure (migration) ± SEM (*n* = 3). Statistical significance was determined using two-way ANOVA with Bonferroni’s post-hoc test (*p* > 0.05: ns, *p* < 0.01: **, *p* < 0.001: *** compared to shLuc).

**Figure 3:**
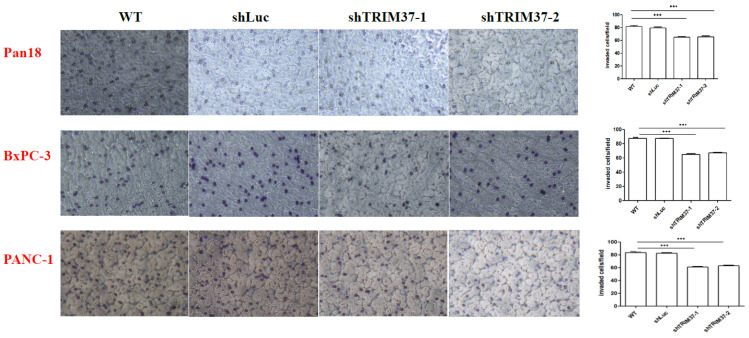
The knockdown of TRIM37 reduced pancreatic cancer cell invasion ability. The invasions were measured using Matrigel invasion assay. Pan18, BxPC-3, and PANC-1 cells (5 × 10^4^) were seeded in the upper chamber for 2 days, and then the invaded cells were counted. Representative images of invaded pancreatic cancer cells (Original magnification, ×20) and quantification of invaded cells are presented. The numbers represent the mean invading cell count ± SEM (*n* = 3). Statistical significance was determined using two-way ANOVA with Bonferroni’s post-hoc test (*p* < 0.001: *** compared to shLuc).

In the wound healing assay, the migration area of pancreatic cancer cells at different time points was dramatically decreased when TRIM37 was knocked down compared with WT and shLuc control groups (Figure 2). Similarly, the results of the invasion assay indicated that the number of invading TRIM37 knockdown pancreatic cancer cells was significantly reduced (Figure 3).

### 2.3. TRIM37 Knockdown Reduced the Colony Formation Capacity of Pancreatic Cancer Cells In Vitro

Our data showed that TRIM37 promoted pancreatic cancer cell migration (Figure 2) and invasion (Figure 3). In order to metastasize and survive in other organs, cancer cells must possess a proliferation ability to form colonies in new places in addition to increased migration and invasion ability. Thus, we examined the influence of TRIM37 knockdown on the ability of pancreatic cells to proliferate indefinitely using colony formation assay.

**Figure 4:**
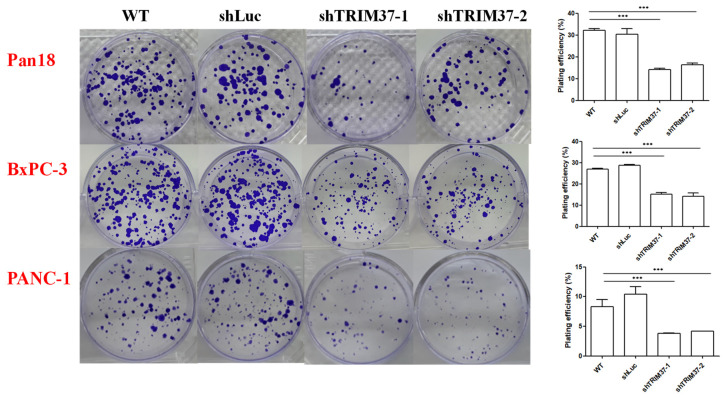
The knockdown of TRIM37 inhibited the clonogenicity of pancreatic cancer cells. Colony formation assay was conducted by seeding 300 cells of Pan18 for 7 days, 500 cells of BxPC-3 for 7 days, and 1000 cells of PANC-1 for 18 days. Representative images of pancreatic cancer colonies and their quantification are presented. The numbers represent the mean colony count ± SEM (*n* = 3). Statistical significance was determined using one-way ANOVA with Bonferroni’s post-hoc test (*p* < 0.001: *** compared to shLuc).

Compared to WT and shLuc control cells, a decrease in the number of colonies was observed in TRIM37 knockdown pancreatic cancer cells (Figure 4). Moreover, the size of TRIM37 knockdown cell clones was smaller than that of WT and shLuc control cell clones.

### 2.4. TRIM37 Facilitated Pancreatic Cancer Progression In Vivo

TRIM37 has been implicated in many types of cancers, and the knockdown of TRIM37 has been shown to reduce the tumor burden in a wide range of cancers, including lung, liver, and colon cancers [28,29,30]. Our in vitro data showed that TRIM37 promoted pancreatic cancer viability, migration, invasion, and clonogenicity. Therefore, we investigated the effect of TRIM37 on pancreatic cancer progression in vivo.

**Figure 5:**
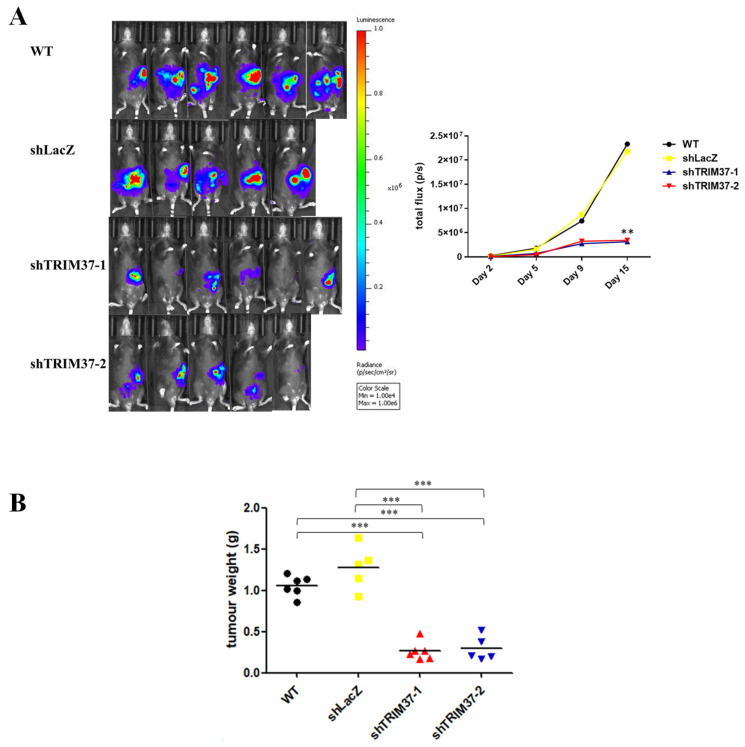
The knockdown TRIM37 of pancreatic cancer suppressed tumor growth in vivo. Pan18-GFP-Luc cells (5 × 10^4^) were injected orthotopically into syngeneic C57BL/6 mice. Tumor growth was monitored using the IVIS spectrum system. (**A**) Images of pancreatic cancer bioluminescence signal on day 15 and quantification of signals during tumor progression. The numbers represent the mean total flux (p/s) (WT: *n* = 6, shLacZ: *n* = 5, shTRIM37-1: *n* = 6, shTRIM37-2: *n* = 5). (**B**) After 17 days, the mice were sacrificed, and the tumors were dissected and weighed. The numbers represent the mean tumor weight ± SEM (WT: *n* = 6, shLacZ: *n* = 5, shTRIM37-1: *n* = 6, shTRIM37-2: *n* = 5). Statistical significance was determined using one-way ANOVA with Bonferroni’s post-hoc test (*p* < 0.01: **, *p* < 0.001: *** compared to WT or shLacZ).

To examine whether TRIM37 could contribute to progression in vivo, an orthotopic syngeneic mouse model was used. Pancreatic cancer Pan18-GFP-Luc WT cells or cells bearing shLacZ or shTRIM37 plasmid, were injected into the pancreas of mice, and tumor development was detected using the IVIS Spectrum System for 15 days. Although all injected pancreatic cancer cells were capable of developing tumors, it was observed that the silencing of TRIM37 in mouse pancreatic cancer cells significantly suppressed the tumor growth (Figure 5A). In addition to the reduction in bioluminescence signals, a significant decrease in the weight of TRIM37 knockdown tumors was observed (Figure 5B).

### 2.5. Knockdown of TRIM37 in Pancreatic Cancer Cells Reduced the Proportion of CD11b^+^F4/80^+^ MHCII^low^ Immunosuppressive Macrophages in the Tumor Milieu, Partly through Regulatory Role of TRIM37 in Cytokine Production

**Figure 6:**
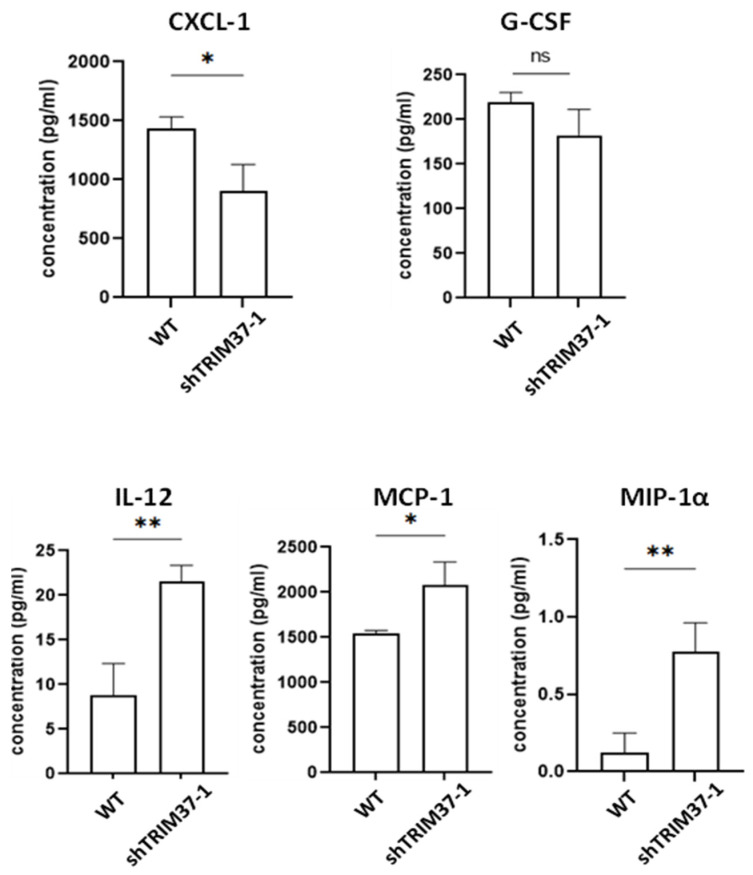
TRIM37 modulated cytokine production by pancreatic cancer cells in vitro. Pan-18 cells (3.5 × 10^4^) were seeded on 6-well tissue culture plates. After the attachment of the cells to the plate, the medium was replaced with fresh medium, and the cells were cultured for another 48 h. The cytokine concentration in the culture medium was measured by using Bio-Plex^TM^ Pro Mouse cytokine 23-plex panel (BioRad, Hercules, CA, USA). The numbers represent the mean ± SEM (WT: *n* = 3, shTRIM37: *n* = 3). Statistical significance was determined using the unpaired Student’s *t*-test (*p* < 0.05: *, *p* < 0.01: **, ns: not significant compared to WT).

TRIM37 contains the TRAF domain that can interact with other proteins possessing the TRAF domain. Proteins containing TRAF domains, such as TRAF proteins, are known to regulate the production of cytokines. Indeed, TRIM37 has been reported to regulate cytokine production [27]. Thus, we examined the effect of TRIM37 knockdown on cytokine production in pancreatic cancer cells. The concentrations of CXCL-1 was significantly decreased in TRIM37 knockdown pancreatic cells, while the levels of IL-12, monocyte chemoattractant protein-1 (MCP-1), and macrophage inflammatory protein (MIP)-1α were increased (Figure 6). In addition, although the level of granulocyte-colony stimulating factor (G-CSF) was not significantly decreased, a trend in downregulation was observed. These data show that TRIM37 can regulate cytokine production in pancreatic cancer cells.

Pancreatic cancer cells secrete mediators, including cytokines, chemokines, and growth factors, to modulate immune cell profiles, thereby promoting cancer progression. Thus, we examined the changes in the immune cell profile in the tumor milieu in vivo.

**Figure 7:**
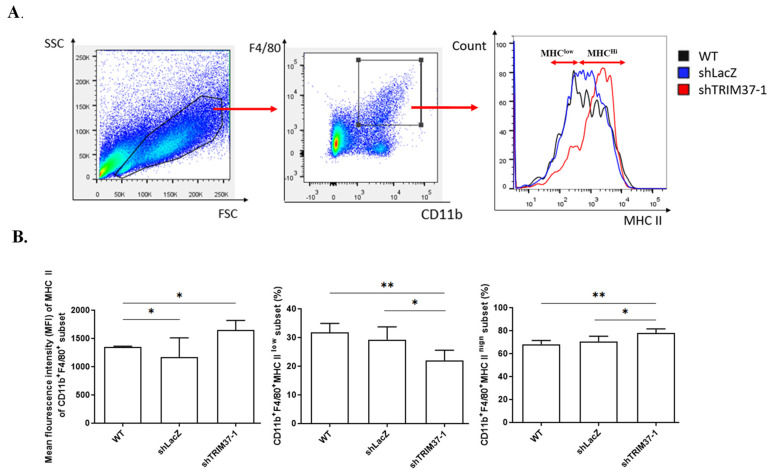
TRIM37 modulated the immune system by increasing the proportion of CD11b^+^F4/80^+^MHCIIlow macrophages in the tumor milieu. Single-cell suspension was isolated from the tumor mass obtained from different groups, and flow cytometry was conducted to analyze the cell population. (**A**) Representative images of the gating strategy of CD11b^+^F4/80^+^MHCIIlow and CD11b^+^F4/80^+^MHCIIhi populations. (**B**) Quantification of mean fluorescence intensity of MHC class II on CD11b^+^F4/80^+^ macrophages (left), percentage of CD11b^+^F4/80^+^MHCIIlow macrophage population (middle), and percentage of CD11b^+^F4/80^+^MHCIIhi macrophage population. The numbers represent the mean ± SEM (WT: *n* = 3, shLacZ: *n* = 3, shTRIM37: *n* = 5). Statistical significance was determined using the unpaired Student’s *t*-test (*p* < 0.05: *, *p* < 0.01: ** compared to WT or shLacZ).

It was observed that the WT and shLacZ tumor groups had significantly lower mean fluorescence intensity of MHC class II on CD11b^+^F4/80^+^ macrophages and a higher percentage of CD11b^+^F4/80^+^MHCII^low^ compared to the shTRIM37-1 tumor group (Figure 7). These results indicate that TRIM37 plays a key role in regulating the immune microenvironment in pancreatic cancer.

## 3. Discussion

Although tremendous development has been made in the field of diagnosis, surgical skills, and treatment, pancreatic cancer is still one of the most lethal malignancies worldwide. Focused research is needed to identify factors that have high value as biomarkers for diagnosing pancreatic cancer at an early stage and potential therapeutics.

In this study, we investigated the role of TRIM37 in regulating pancreatic cancer progression. Numerous in vitro experiments have been conducted to explore the effect of TRIM37 knockdown on the development and progression of pancreatic cancer. Our results showed that TRIM37 knockdown significantly inhibited the viability of pancreatic cancer cells. The knockdown of TRIM37 in mouse pancreatic cancer cell line Pan18 and human pancreatic cancer cell lines, PANC-1 with *KRAS* mutation and BxPC-3 with wild-type *KRAS*, resulted in a significant reduction in the viability of pancreatic cancer cells. This finding is consistent with previous studies [16,25,28,31,32], demonstrating that TRIM37 promotes the proliferation and progression of many types of cancer, including non-small cell lung cancer, colorectal cancer, and osteosarcoma.

The number of centrioles was reduced via the ubiquitination function of TRIM37 by suppressing the positive regulator of centriole reduplication, thereby leading to chromosome segregation errors [33]. As a result, TRIM37 inhibited the cell cycle. Moreover, through monoubiquitylation of PEX5, a receptor of peroxisomal targeting signals, TRIM37 enhances PEX5 stability, which in turn increases the import of peroxisomal proteins [34]. The deletion of TRIM37 or PEX5 results in errors in peroxisomal matrix protein import, resulting in increased cell apoptosis [34]. TRIM37 also activates mTORC1 signaling pathway, which promotes cell growth and inhibits autophagy. Indeed, TRIM37 mutation enhanced autophagy through the inhibition of the mTORC1/TFEB pathway, resulting in TFEB translocation into the nucleus to activate genes involved in liposome biogenesis and autophagy [35]. As previously mentioned, TRIM37 is the only member of the TRIM family that has the TRAF domain. TRIM37 has been reported to interact with TRAF2 and TRAF6 to promote cancer cell proliferation and chemoresistance. Therefore, we proposed that TRIM37 greatly contributes to pancreatic cancer cell proliferation.

Metastasis is responsible for the recurrence of cancer; therefore, it is imperative to find effective strategies to restrain tumor dissemination. Meanwhile, patients with pancreatic cancer have a high rate of metastasis at the time of diagnosis. Cell migration and invasion are critical steps for cancer cells to metastasize. Our results show that TRIM37 promoted pancreatic cancer cell migration, invasion, and clonogenicity. A decrease in the expression of TRIM37 in both mouse and human pancreatic cancer cells resulted in decreased invasion and migration ability of cells. The number of colonies formed by pancreatic cancer cells following TRIM37 knockdown was reduced; additionally, these colonies had a smaller size than that of the control colonies.

It has been demonstrated that TRIM37 facilitates the invasion and metastatic ability of cells in gastric cancer and hepatocellular carcinoma via activation of the epithelial-mesenchymal transition (EMT) and Wnt/β-catenin signaling pathway, respectively [18,29]. The metastatic ability of lung cancer cells was inhibited by the knockdown of TRIM37, as Akt activity was suppressed; thereafter, lung cancer cells were prone to apoptosis [30]. In addition, lung cancer cells were less likely to migrate and proliferate after the suppression of TRIM37 either through EMT process inhibition of oncogenic transcriptional factor downregulation [17]. Similarly, the survival and migration of pancreatic cancer cells were promoted by TRIM37 [32]. Furthermore, the overexpression of TRIM37 enhanced the growth and migration of pancreatic cancer cells by activating the β-catenin/TCF signaling pathway [32]. These findings suggest that TRIM37 can promote cancer metastasis.

To examine the impact of TRIM37 on the progression of pancreatic cancer in vivo, we injected mouse pancreatic cancer cells containing shTRIM37 or shLacZ plasmid or WT into syngeneic mice. TRIM37 knockdown significantly inhibited tumor growth, as the bioluminescence signal in the TRIM37 knockdown group was dramatically reduced compared to that in the control groups. In addition, the tumor weight was decreased considerably in the TRIM37 knockdown group. In terms of tumor metastasis, we observed that the number of tumors formed in the peritoneum of WT and shLacZ control groups was higher than that in the TRIM37 knockdown group (data not shown), demonstrating that TRIM37 plays a positive role in regulating cell metastasis.

TRIM37 promoted lung cancer progression by activating the PI3K/AKT and NF-κB pathways, and TRIM37 suppression resulted in significant decreases in tumorigenesis in vivo [25,30]. Similarly, TRIM37 knockdown in colorectal carcinoma inhibited tumor growth [28]. In hepatocellular carcinoma, TRIM37 activated the Wnt/β-catenin pathway from promoting metastasis, and the overexpression of TRIM37 increased the number of lung metastatic nodules in vivo [29].

After investigating the immune cell population in tumor milieu, we observed that tumors with TRIM37 knockdown had significantly lower numbers of CD11b^+^F4/80^+^MHCII^low^ macrophages.

Previous studies have shown that pancreatic cancer increases the secretion of a plethora of cytokines, chemokines, and growth factors to attract neutrophils, as well as macrophages and myeloid-derived suppressor cells (MDSCs) [36,37,38]. In colorectal cancer, an increase in the CD11b^+^F4/80^hi^MHCII^low^ macrophage subpopulation was observed during cancer progression, as cancer cells release high amounts of CSF1, a crucial growth factor for macrophage proliferation and survival [39]. Both macrophage subpopulations expressed enhanced arginase-1 (ARG1) transcription, a marker for M2-polarized macrophages and tumor-associated macrophages (TAM); however, CD11b^+^F4/80^hi^MHCII^low^ was the major source of Arginase1 in the tumors. In another study on hepatocellular carcinoma, two distinct TAM subsets were found to coexist within the tumor milieu, with the MHC class II^hi^ appearing in the early phase of tumor development and was associated with tumor suppression, while the MHC class II^low^ was prominent during tumor progression [34]. MHC class II^low^ TAM increases the production of IL-10 and TGF-β to inhibit T cell proliferation and the secretion of MMP-9 and VEGF to promote tumor metastasis [40].

In the present study, the knockdown of TRIM37 suppressed the production of CXCL-1, whereas it enhanced the secretion of IL-12, MCP-1, and MIP-1α. Meanwhile, although the knockdown of TRIM37 did not significantly suppress the production of G-CSF, we observed a trend in downregulation. G-CSF/G-CSFR promoted the development of pro-tumoral macrophages in colon and pancreatic cancer [41]. Patients with breast cancer have high levels of G-CSF, which supports M2-like macrophages, and anti-G-CSF treatment significantly reduced the proportion of MHCII^low^ blood monocytes and TAM, as well as lung metastasis [42]. CXCL1 and CXCL2, highly expressed in several tumor cells, increase the generation of monocytic MDSC that inhibits T cell proliferation [43]. In contrast to the effect of TRIM37 knockdown in pancreatic cancer on CXCL-1 and G-CSF, there was an increase in the production of IL-12, MCP-1, and MIP-1α. IL-12 increases antigen processing and presentation ability by professional antigen-presenting cells, including CD11b^+^F4/80^hi^ macrophages and CD11b^+^CD11c^+^MHCII^hi^ dendritic cells, and suppresses tumor growth in melanomas [44]. In pancreatic cancer, the infiltration of monocytes and macrophages to degrade fibrosis is dependent on MCP-1 [45]. MIP-1α can enhance antitumor activity by recruiting natural killer (NK) cells to enhance dendritic cell infiltration and support T cell function [46].

In summary, the data obtained from both in vitro and in vivo experiments indicate that TRIM37 plays an oncogenic role in the progression of pancreatic cancer. Furthermore, TRIM37 in pancreatic cancer cells could modulate the immune profile by increasing the proportion of immunosuppressive macrophages in the tumor microenvironment to promote tumor aggressiveness.

## 4. Materials and Methods

### 4.1. Cell Lines

The mouse cell line Pan18-GFP-Luc (Pan18) was kindly provided by Dr. Chia-Ning Shen from Academia Sinica, Taiwan [47], and cultured in Dulbecco’s modified Eagle’s medium (DMEM) supplemented with 10% heat-activated fetal bovine serum (FBS), 100 U/mL of penicillin, 100 μg/mL of streptomycin, 2 nM of L-glutamine, 0.1 nM of non-essential amino acid, and 1 nM of sodium pyruvate. Human pancreatic ductal cell line PANC-1, with *KRAS*, *TP53* and *CDKN2A/p16* mutations was purchased from the Food Industry Research and Development Institute, Taiwan, and cultured in DMEM supplemented with 10% heat-activated FBS, 100 U/mL of penicillin, 100 μg/mL of streptomycin, and 2 nM of L-glutamine. Human pancreatic cancer cell line BxPC-3 with *KRAS* wild type (WT) and *TP53*, *CDKN2A/p16* and *SMAD4/DPC4* mutations was purchased from the Food Industry Research and Development, Taiwan, and cultured in Roswell Park Memorial Institute (RPMI) 1640 medium supplemented with 10% heat-activated FBS, 100 U/mL of penicillin, 100 μg/mL of streptomycin, and 2 nM of L-glutamine. The cells were incubated at 37 °C in a humidified incubator under 5% CO_2_.

### 4.2. Animals

Female C57BL/6 mice (6–8 weeks old) were purchased from the National Laboratory Animal Center of Taiwan. All experiments in this study were conducted in accordance with the Institutional Animal Care and Use Committee of China Medical University; and followed the protocols of the Care of the animals and surgical procedures of China Medical University (Approval protocol number: 2017-371).

### 4.3. Antibodies for Western Blotting and Flow Cytometry

Anti-TRIM37 (N1N2) and Anti-β-actin (AC15) antibodies were purchased from GeneTex (Hsinchu City, Taiwan) and Abcam (Boston, MA, USA), respectively. Alexa Flour^®^ 488 Rat Anti-Mouse CD11b (M1/70) and PE Cy7 Hamster Anti-Mouse CD11c (HL3) antibodies were purchased from BD Pharmingen™; PE anti-mouse F4/80 Antibody (BM8) antibodies were purchased from BioLegend, and APC MHC class II antibody (M5/114.15.2) was purchased from eBioscience™.

### 4.4. Generation of TRIM37 Knockdown Cells

Commercial lentivirus bearing TRIM37 short hairpin RNA (shRNA), and shLuc and shLacZ were purchased from the National Core Facility for Manipulation of Gene Function by RNAi, miRNA, miRNA sponges and CRISPR/Genomic Research at Academia Sinica, Taiwan. TRIM37 knockdown cells were generated by infecting the cells with lentivirus bearing TRIM37 shRNA. Virus infection was performed according to the manufacturer’s protocol. Briefly, the cells were seeded one day before infection, and the virus with a multiplicity of infection (MOI) = 3 was added into the growth medium with 8 μg/mL polybrene, and then cells were centrifuged at 12,000× *g* for 30 min at 37 °C. Puromycin was used for the selection of knockdown cells.

### 4.5. Western Blotting

Total cell lysates were prepared in sodium dodecyl sulfate (SDS) lysis buffer supplemented with the protease inhibitor cocktail. Equal amounts of protein (10 μg) were separated on 8% SDS-PAGE and then transferred to polyvinylidene fluoride membrane. Afterward, the membranes were incubated for 1 h in tris-buffered saline with 0.05% Tween-20 (TBST) with 5% milk, followed by incubation with the primary antibody at 4 °C overnight. After washing the membrane, the secondary antibody was added. The bands were detected using the enhanced chemiluminescence substrate, and the images were quantified using the ImageJ 1.52k software.

### 4.6. MTT Assay

Cells (Pan18 and BxPC3: 4 × $10^{3}$, PANC-1: 6 × $10^{3}$) were plated in 96-well plates for 24, 48, 72 and 96 h. One hundred microliters of medium containing 10 μL of 5 mg/mL MTT solution dissolved in water was added to each well and incubated for 60 min. The formazan crystals formed were dissolved in DMSO. Absorbance was measured at 570 nm.

### 4.7. Wound Healing Assay

Cells were seeded in 12-well plates until they reached 90% confluence. A wound was generated by scraping the plates. Images of the wounded monolayer were captured at different time points, and cell migration was quantified using the ImageJ 1.52k software. The data were obtained by calculating the percentage of the migration area of the cells to the area of the primary wound.

### 4.8. Matrigel Invasion Assay

The cell invasion assay was performed using 6.5 mm Transwell^®^ with 8 μm pores. In brief, the upper chamber was pre-coated with 100 μL medium containing 0.1 μg Matrigel and allowed to air-dry overnight at room temperature. The cells were resuspended in the medium supplemented with 1% FBS and added to the upper chamber; the medium containing 10% FBS was added to the lower chamber. After 48 h of incubation, the non-migrated cells were removed, and the filters containing invaded cells were fixed in 4% paraformaldehyde and stained with 0.5% crystal violet. Images from ten random fields of each membrane were captured, and the number of invaded cells was counted.

### 4.9. Colony Formation Assay

The cells were seeded in 6-well plates, and the medium was replaced every alternate day. After culturing Pan18 and BxPC-3 for 7 days and PANC-1 for 18 days, the cells were fixed in 4% paraformaldehyde and stained with 0.5% crystal violet. Colonies with more than 50 cells were counted manually. Plate efficiency (PE) was measured using the following formula:

$$\mathrm{PE}=\frac{Number\;of\;colony\;counted}{Number\;of\;cells\;plated}\times 100$$

### 4.10. Orthotopic Pancreatic Cancer Mouse Model

Pan18-GFP-Luc cells (5 × 10^4^ cells) were mixed with Matrigel and injected into the pancreas of mice. Tumor growth was measured twice a week by measuring the tumor bioluminescence by intraperitoneal injection of D-luciferin potassium salt dissolved in PBS at a dose of 15 mg/kg into the mice 5 min before imaging. Animal bioluminescent imaging was conducted using an in vivo imaging system (IVIS). The region of interest was selected, and the radiance and total flux values were measured using the Living Image^®^ 4.5.5 software (PerkinElmer, Akron, OH, USA). After 17 days, the mice were sacrificed, and the tumors were dissected and weighed.

### 4.11. Multiplexed ELISA Analysis

Pan-18 cells (3.5 × 10^5^) were seeded on 6-well tissue culture plates. After the attachment of the cells to the plate, the medium was replaced with fresh medium, and cells were cultured for another 48 h. The cytokine concentration in the culture medium was measured by using Bio-Plex^TM^ Pro Mouse cytokine 23-plex panel (Bio-Rad, Hercules, CA, USA). The data was performed in triplicate according to the manufacturer’s instructions on a Bio-Plex™ system (Luminex Bio-Plex™ 200 System, Bio-Rad).

### 4.12. Flow Cytometry

The tumor mass was minced into small pieces and digested with Hank’s balanced salt solution (HBSS) containing 1 mg/mL collagenase I (300 U/mL) and 0.02 mg/mL DNase I. The digested tissue was mashed to obtain single-cell suspension. RBC lysis buffer was used for the depletion of erythrocytes, and 10^6^ cells per tumor were blocked with FcR blocking reagent for 10 min. The cells were incubated with a surface antibody mixture for 30 min at 4 °C in the dark. The samples were analyzed using BD FACS Canto, and the proportion of each cell population was analyzed using the FlowJo v10.0.7.

### 4.13. Statistical Analysis

GraphPad Prism 5 was used for statistical analysis. Unpaired Student’s *t*-test was used to compare two groups, and one-way or two-way analysis of variance with Bonferroni’s post-hoc test was used for multiple comparisons. *p*-values of less than 0.05 were considered statistically significantly (*p* < 0.05: *, *p* < 0.01: **, *p* < 0.001: ***).

## 5. Conclusions

In conclusion, our study demonstrated that knockdown of TRIM37 reduced the viability, migration, and invasion of pancreatic cancer cells both in vitro and in vivo and regulated cytokine production. These results suggest that TRIM37 facilitates the progression of pancreatic cancer. In addition, TRIM37 could increase the proportion of pro-tumor macrophages to enhance tumor progression. Therefore, understanding the mechanism by which TRIM37 facilitates pancreatic cancer progression could provide insight into potential targets for the development of an effective therapeutic strategy against this malignant disease. However, further basic and clinical investigations are required to confirm this.

## Data Availability

The data that support the findings of this study are available from the corresponding author upon reasonable request.

## Supplementary Materials

The following supporting information can be downloaded at: https://www.mdpi.com/article/10.3390/ijms23031176/s1.
