# Supplementary material for: TRIM37 Promotes Pancreatic Cancer Progression through Modulation of Cell Growth, Migration, Invasion, and Tumor Immune Microenvironment
Source: Int J Mol Sci. 2022 Jan 21;23(3):1176. doi: 10.3390/ijms23031176 (PMC8835669; doi:10.3390/ijms23031176)
Supplement: Supplementary file 1 [file ijms-23-01176-s001.zip › ijms-1465706-supplementary.pdf]

Supplementary Material:

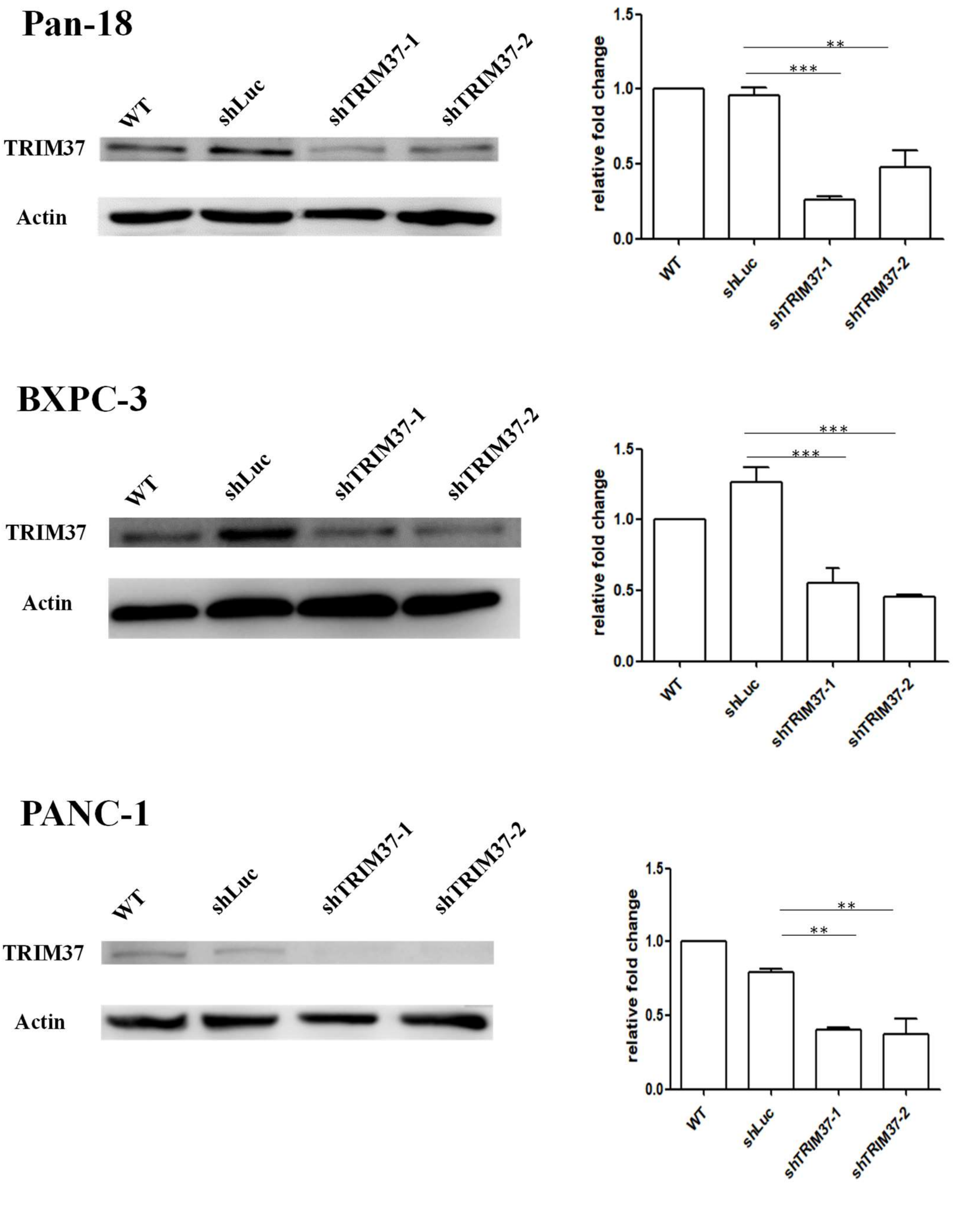

Supplemental Figure S1: *shTRIM37s reduce the protein expression of TRIM37 in pancreatic cancer cells*

The western blot was performed to measure TRIM37 protein level in Pan18, BxPC-3 and PANC-1 after infected with lentivirus containing shTRIM37 or shLuc plasmid. The expression level of TRIM37 was normalized to ß-actin using Image J software. The relative fold change defines the level of relative expression compared to that of WT group. Numbers were represented the mean of relative fold change ± SEM (n=3). Statistical significance was determined using one-way ANOVA with the Bonferroni's post-test ($p<0.01$: **, $p<0.001$: *** compared to shLuc).
